# Determining diagnostic delays in Romanian multiple myeloma patients using the Aarhus statement

**DOI:** 10.3389/fmed.2024.1372907

**Published:** 2024-05-24

**Authors:** Ruxandra Irimia, Sorina Badelita, Sinziana Barbu, Ioana Loredana Cirlan, Larisa Zidaru, Daniel Coriu

**Affiliations:** ^1^Department of Hematology, Carol Davila University of Medicine and Pharmacy, Bucharest, Romania; ^2^Fundeni Clinical Institute, Bucharest, Romania

**Keywords:** diagnostic delays, diagnostic errors, multiple myeloma, Aarhus statement, diagnostic intervals

## Abstract

**Introduction:**

Multiple Myeloma (MM) is classified as one of the most challenging cancers to diagnose, and the hematological malignancy is associated with prolonged diagnostic delays. Although major steps have been made in the improvement of MM patient diagnosis and care, Romanian patients still face long diagnostic delays. Thus far, there have been no studies evaluating the factors associated with diagnostic errors in Romanian MM patients.

**Methods:**

Using the Aarhus statement, we prospectively determined the diagnostic intervals for 103 patients diagnosed with MM at Fundeni Clinical Institute, between January 2022 and March 2023.

**Results:**

Our data revealed that the main diagnostic delays are experienced during the “patient interval.” Patients spend a median of 162 days from the first symptom onset until the first doctor appointment. Bone pain is the most frequently reported symptom by patients (78.64%), but it leads to a medical-seeking behavior in only half of the reporting patients and results in a median delay of 191 days. The changes in routine lab tests are considered most worrisome for patients, leading to a medical appointment after a median of only 25 days. The median primary care interval was 70 days, with patients having an average of 3.7 medical visits until MM suspicion was first raised. The secondary care interval did not contribute to the diagnostic delays.

**Discussion:**

Overall, the median diagnostic path for MM patients in Romania was more than 6 months, leading to a higher number of emergency presentations and myeloma-related end-organ damage.

## Introduction

Diagnostic errors, defined as delayed, missed, or wrong diagnoses, are considered to be the leading cause of patient harm (1). Diagnostic errors are recognized as a top priority challenge in public health by the World Health Organization and the Agency for Healthcare Research and Quality (AHRQ).

A recent analysis of malpractice claims has identified cardiovascular events, cancers, and infections as the top frequently misdiagnosed conditions associated with serious harm to patients (2–4).

Multiple Myeloma (MM) is classified as one of the most challenging cancers to diagnose. It is characterized by prolonged diagnostic delays and accounts for half of all the premature deaths caused by hematological malignancies (5–7).

The causes of long delays in diagnosis are not yet fully understood but could include disease-specific factors such as the insidious onset, lack of symptom specificity, and absence of definitive “red flags” (8–11). While disease-specific factors contribute significantly to diagnostic delays, healthcare-related factors also play a pivotal role. These include inadequate recognition of key symptoms, misinterpretation of hematological and biochemical abnormalities, and barriers to accessing care (7, 12). Furthermore, the patients’ healthcare-seeking behavior, health literacy, or awareness of MM can further contribute to the delays (11).

The typical onset of MM is gradual, often presenting symptoms such as fatigue and persistent bone pain, that can be easily misinterpreted, particularly in older patients or in those with comorbidities (7). In addition, the classical manifestations of MM, such as hypercalcemia, renal failure, anemia, and bone lesions, rarely occur simultaneously at diagnosis (12).

Successful management of MM patients relies on an accurate and timely diagnosis. Unfortunately, one-third of MM patients are diagnosed in the emergency department (ED) at an advanced stage, leading to poor outcomes (5, 12). Emergency presentations often include spinal cord compression syndrome, bone fractures, anemia, sepsis, and neurological impairment, resulting in increased morbidity and mortality (13).

Although Romania has made major steps toward improving the care of cancer patients, the diagnosis of MM remains a major problem.

According to Globocan, in 2020, more than 880 new cases of MM were estimated to have been diagnosed in Romania. However, only 550 new patients have been recorded in the national registries, over 40% being diagnosed at a late ISS stage III (14–16). The discrepancy between expected and recorded MM cases in Romania highlights potential issues in the diagnostic pathway, including missed or incorrect diagnoses.

It is important to mention that in Romania, patients can perform “on-demand” lab tests without a prior medical visit. Furthermore, private clinics offer appointments to specialist physicians without a referral from the primary care physician. The fragmented nature of the medical appointments and lab evaluations may contribute to diagnostic delays and inadequate assessments.

Our study is the first assessment of the diagnostic paths of MM patients in Romania. Using the Aarhus statement as a framework (15), we aimed to document diagnostic intervals and identify primary causes of delays. Additionally, our research provides insight into the various routes leading to the final MM diagnosis, shedding light on missed opportunities for timely diagnosis.

## Methods

### Study design

A prospective, unicentric study was conducted at Fundeni Clinical Institute, Romania’s main tertiary hematology center. The aim of the study was to evaluate the diagnostic paths of MM patients in Romania and identify factors associated with diagnostic delays.

### Participant selection

The study was approved by the institution’s ethics committee board (approval no. 14527/Jan 2022), and all patients provided written informed consent according to local regulations before enrolling. A total of 106 adult patients who received an MM diagnosis between January 2022 and March 2023 were initially evaluated. Three patients were excluded from the study due to critical conditions and subsequent mortality. The remaining 103 patients were considered eligible.

### Data collection

**Figure 1:**
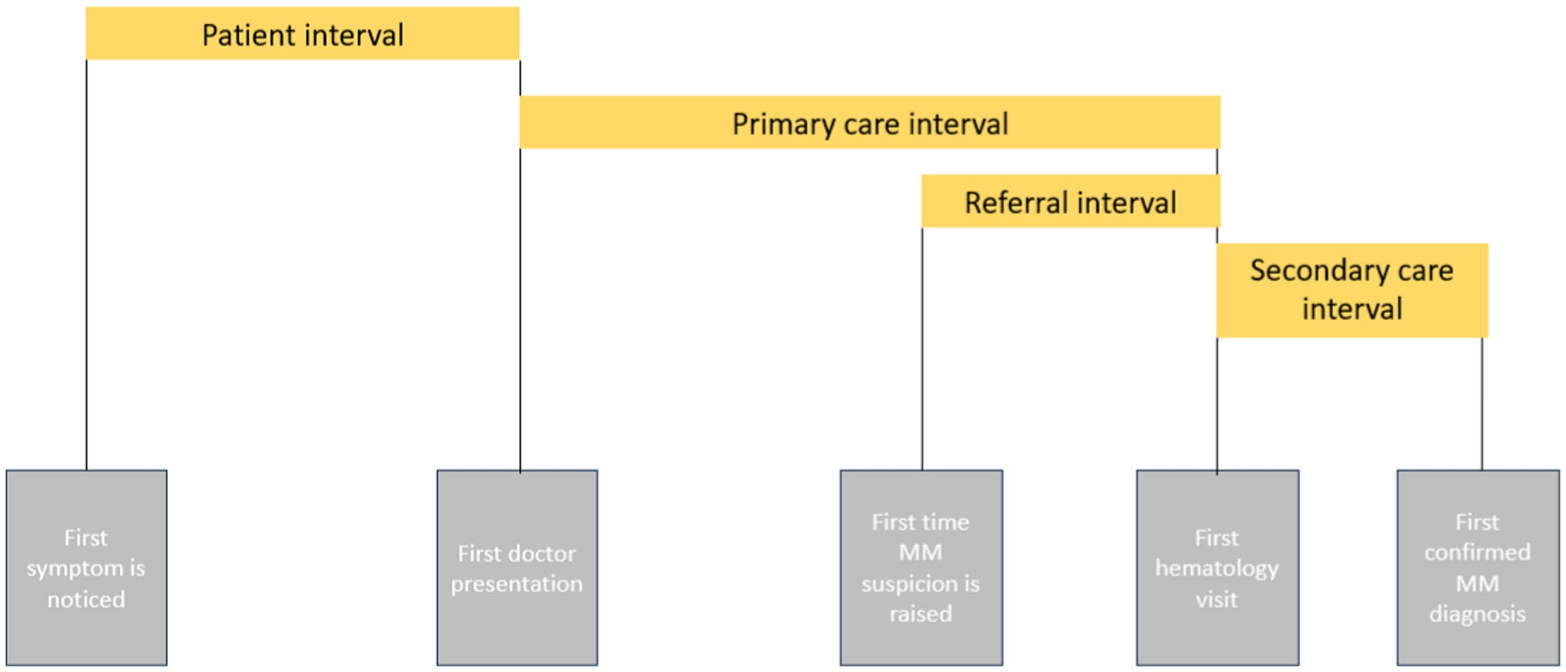
Diagnostic intervals according to the Aarhus statement (17, 18).

During the initial admission, a structured questionnaire was used to interrogate the main stages of the cancer diagnostic path, as defined by the Aarhus statement (17, 18). This included the patient interval (days from symptom onset to the first doctor presentation), primary care interval (days from the first doctor visit to the first hematologist referral), and secondary care interval (days from the first hematology visit to confirmation of MM diagnosis). Since the healthcare system in Romania allows “on-demand” appointments with specialists without prior primary care consultation, the primary care interval was further divided into first doctor appointment, i.e., when first-time suspicion was raised and first referral to a hematologist (Figure 1).

Based on the literature review and discussions with myeloma specialists, we defined the possible key symptoms prompting action as “bone pain,” “bone fractures,” “fatigue,” “pallor,” “weight loss,” “nausea,” “frequent infections,” and “paresthesia,” but also covered incidental discoveries reflected by changes in “on-demand routine lab tests” [hemoglobin (Hb), creatinine, erythrocyte sedimentation rate (ESR), calcium or serum electrophoresis]. We evaluated the diagnosis route, such as whether patients were first evaluated by a primary care physician (family doctor) or by a specialist doctor (internal medicine, nephrology, radiologist, neurologist, neurosurgeon, etc.). The number of visits to doctors before the first suspicion of MM was also recorded.

Each questionnaire was completed by the study team during the initial diagnostic assessment, based on the patient history, a detailed interview, and a thorough evaluation of previous medical visits and lab reports. The quality control measures, including audits of completed questionnaires and data entry verification, were implemented to maintain data quality and consistency.

### Statistical analysis

Four main different statistical tests (Kolmogorov–Smirnov, Kolmogorov–Smirnov–Lilliefors Corr., Shapiro–Wilk, and Anderson-Darling) were used to test the normal distribution of the data. As the data were non-normally distributed, the non-parametric Mann–Whitney test was employed for comparisons. Diagnostic delays between intervals were calculated using DataTab software. Statistical significance was defined as a *p*-value of <0.05.

## Results

Our study included 103 adults with newly diagnosed MM. Among them, 51 patients (49.51%) were men and 52 (50.42%) were women, with a median age of 62 years. Furthermore, 62 patients (60.19%) resided in an urban area, while 41 (39.81%) were located in a rural area.

### Patient interval

The delays in the patient interval are often a result of the patient’s failure to recognize cancer symptoms.

In our study, the symptoms experienced by patients were highly heterogeneous and unspecific.

The most commonly reported symptoms were bone pain (78.64%), fatigue (57.28%), unexplained weight loss (13.59%), and pale appearance (25%).

However, patients often did not perceive these symptoms as alarming until they interfered with their daily activities. This is concordant with existing reports stating that patients would often interpret the fatigue associated with anemia as a result of day-to-day activities. In an older population, myeloma-related bone pain can be overseen as it may be perceived to occur on a previous background of rheumatic or degenerative skeletal pain (11, 19).

Despite the high prevalence, bone pain led to a medical presentation in only 59.02% of the patients, and the median time from occurrence to the first doctor visit was 191 days (range 1–1,598 days).

From the total study population, 6.79% of the patients reported not having any prior symptoms and being alerted incidentally only by changes in their on-demand lab test results. Overall, in our study group, 66.02% of patients showed changes in the on-demand routine lab test results, irrespective of whether they were associated with other key symptoms.

The changes in the on-demand lab test results (Hb, creatinine, ESR, calcium, or serum electrophoresis) resulted in 23.3% of patients seeking medical advice, with a statistically significant shorter median time from occurrence to first doctor visit (25 days, range 0–647 days, *p* = 0.005). Furthermore, these changes led to an immediate medical visit in 15.32% of the patients.

In particular, despite experiencing activity-limiting fatigue, a pale appearance, or weight loss, the patients required a median of 172 days (range 5–339 days) until performing on-demand routine lab tests that led to a doctor appointment.

Overall, the median duration of the patient interval was 162 days (range 0–1,128 days), with no differences observed in the patient interval between male and female patients (median 163 days versus 167 days respectively, *p* = 0.234). Using the 75th percentile as a cutoff for the patient interval, we found that 12.62% of patients experienced a delay beyond the threshold of 455 days.

**Figure 2:**
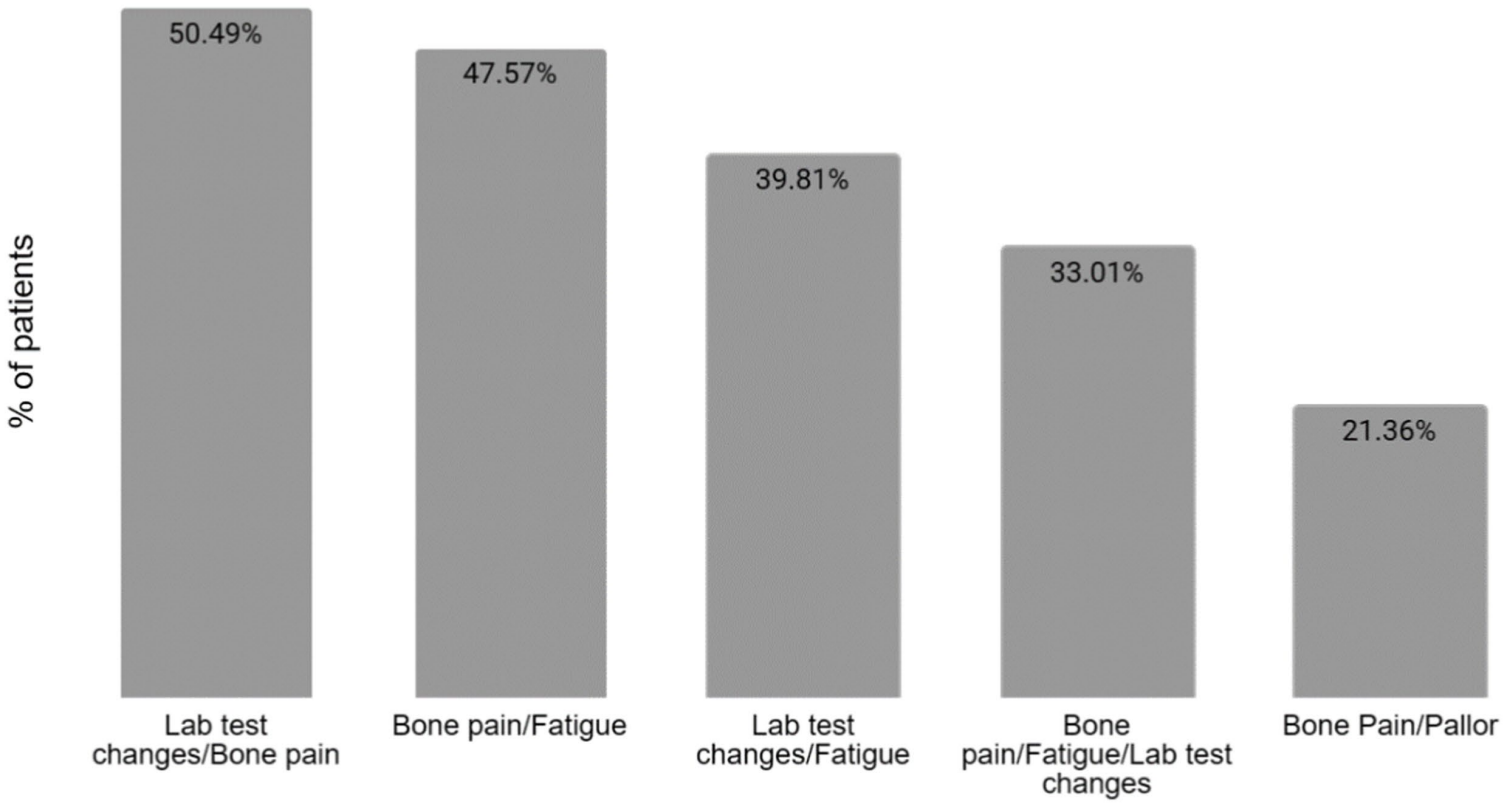
Combinations of manifestations at the time of diagnosis. The most common combination of manifestations at the time of diagnosis is between bone pain and laboratory test changes, in 50.49% of patients, followed closely by the association of bone pain and fatigue in 47.57% of the patients. Only one-third of patients presented with the association between bone pain, fatigue, and changes in the lab tests.

All the reported symptoms are detailed in Table 1. The most frequent combinations of manifestations at the time of diagnosis are detailed in Figure 2.

**Table 1:** Patient demographics and main complains at the time of diagnosis.

1. Patient characteristics	
Age median (range) (years)	64 (42–89)
Sex	
Male	51 (49.51%)
Female	52 (50.49%)
Residence	
Urban	62 (60.19%)
Rural	41 (39.81%)
2. Complaints at diagnosis	
Bone pain	81 (78.64%)
Fatigue	59 (57.28%)
Pallor	26 (25.24%)
Weight loss	14 (13.59%)
Paresthesia	13 (12.62%)
Fractures	7 (6.79%)
Frequent infections	7 (10.14%)
Changes in lab tests	68 (66.02%)

### Primary care interval

The delays in the primary care interval, defined as the duration between the first medical visit to the first time myeloma is suspected, typically arise due to the failure of the primary care physician (family physician/general practitioner) to recognize the myeloma-associated symptoms and to suggest and interpret the right diagnostic tests (19).

In our study, only 42.71% of the patients were referred first to their primary care physician concerning their symptoms.

More than 25% of the patients had an emergency first presentation. Furthermore, 17.47% were first referred to the Emergency Care Unit, while 11.65% had a first appointment with a neurosurgeon presenting with either debilitating vertebral pain or spinal cord compression syndrome.

In addition, 28.17% of patients were first evaluated by a nephrologist due to renal failure associated with MM, while 10.67% were first evaluated by another specialist (rheumatologist, radiologist, neurologist, or internal medicine doctor).

Out of the patients that were first referred to their primary care physician (family physician/general practitioner), 90.29% had been referred to at least one more specialist physician before the suspicion of Multiple Myeloma was raised. Patients who initially consulted their primary care physician had a significantly shorter primary care interval compared to those with other diagnostic routes (median 26 days versus 93 days, *p* = 0.004). On average, patients made 3.7 medical visits (range 2–16) before MM suspicion was raised, with 18.44% requiring five or more visits. The median interval from the first doctor visit to suspicion of myeloma was 66.5 days (range 0–1,077 days). Using the 75th percentile as a cutoff for the primary care interval, we found that 41.75% of patients experienced a delay beyond this threshold.

**Figure 3:**
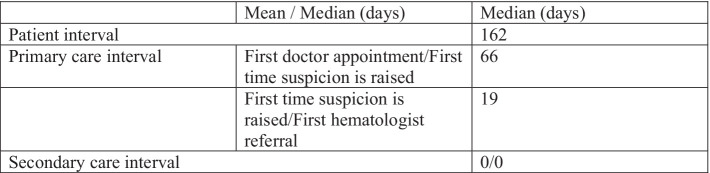
Diagnosis intervals according to the Aarhus statement.

The median time between when the myeloma suspicion was raised and their first visit to our center was 19 days (Figure 3).

### Secondary care interval

In the secondary care interval, defined as the number of days from the first hematology visit to confirmation of MM diagnosis, delays occur due to the physician’s inability to diagnose the disease and to initiate the proper treatment.

Delays in the secondary care interval are typically minimal in tertiary care facilities equipped with modern diagnostic tools. At Fundeni Clinical Institute, where specialized MM management units are established, diagnosis confirmation is prompt and accurate. Upon admission, all patients received a diagnosis during their first admission, with a median duration of zero days. For eligible patients, treatment initiation occurred within a maximum of 72 h. In particular, 31.06% of patients required emergency medical care, primarily due to acute kidney failure, severe hypercalcemia, spinal cord compression, or severe anemia. The majority (60.19%) were diagnosed with ISS stage III MM, with 15.53% requiring hemodialysis.

## Discussion

Our study represents the first-ever analysis of the pathways and delays in the diagnosis of Romanian Multiple Myeloma patients. Our findings reveal that the most significant delays occur during the patient interval, with a median duration of 162 days. This duration aligns closely with the 163-day interval reported by Howell et al. (9) in the UK population. The possible causes for the prolonged patient interval include the unspecific symptoms, with insidious onset, leading patients to overlook or “normalize” them.

It is considered among the general population that cancer should lead to either worrisome or debilitating symptoms. However, in Multiple Myeloma there are no overt “red flags” that can trigger the medical-seeking behavior. Furthermore, classical symptoms such as bone pain might go unnoticed in an elderly population accustomed to chronic pain, while fatigue can often be attributed to anemia or routine daily activities.

The UK population cohort study, performed by Howell et al. (11), shows similar results. They report that many patients experience a phase of “normalization,” during which changes in their health status are often attributed to factors such as aging, minor injuries, recurrence of a previous health issue, or side effects of medication.

Although the most frequently encountered complaints were bone pain and fatigue, our study highlights that changes in on-demand lab tests are the most significant triggers for seeking medical assistance. This may be attributed to the healthcare system’s structure, where the “on-demand” lab tests can be performed without a prior medical visit, either routinely or as a result of a change in the individual’s perception of their general health.

In addition, factors such as poor medical education, shame, financial constraints, and fear of cancer diagnosis contribute to delaying behavior in seeking medical assistance. Consequently, as a direct result of the delays in the patient interval, over 25% of MM patients in Romania have their first medical presentation as an emergency. This finding is similar to the 20% rate of emergency presentations reported in the UK population by Howell et al. (11).

The primary care interval represents a critical stage where missed opportunities can lead to harm for MM patients (20). Myeloma is considered one of the most challenging cancers to suspect and diagnose. Challenges in this stage arise from factors such as the rarity of MM, lack of readily available testing methods, and non-specific presentations (5–7, 20, 21).

Studies conducted in the US medical health system by Ailawadhi et al. (22) have shown that up to a third of MM patients face errors in diagnosis in the primary care setting, with classical signs of MM missed in up to 5% of the patients.

In our study, the primary care interval reflected the complex pathways that lead to the diagnosis of Multiple Myeloma in Romania. Typically, patients seeking evaluation by specialists in the public health sector require a referral from a family physician. However, it is important to mention that in Romania, patients can directly access specialist doctors in the private sector without prior primary care visits, leading to fragmented care and delayed diagnosis. In our study, the data generated emphasizes the pivotal role of primary care physicians in minimizing delays in diagnosis. Patients who sought consultation with their primary care physician experienced markedly shorter primary care intervals compared to those following alternative diagnostic pathways.

Although the diagnostic delays in this interval were not as prolonged as those in the patient interval, they still resulted in delays exceeding 2 months.

Less than half of the patients first consulted their primary care physician, but nearly all of them required at least one additional appointment until the suspicion of Multiple Myeloma was raised.

More concerning is the fact that, on average, the Romanian patient required more than three medical appointments until the suspicion of Myeloma was raised, and over 18% of the patients had 5 or more different prior medical visits. This is consistent with the results reported by Lyratzopoulous et al. (19) showing that more than half of the MM patients in England required three or more prior general practitioner visits before being referred to a hematologist. While less than 10% of breast cancer patients needed multiple medical visits before diagnosis, Multiple Myeloma patients required the highest number of medical consultations (19).

This is particularly relevant since the number of consultations is a strong determinant of the diagnostic delays associated with primary care (23).

The consequences of delays in the initial diagnostic intervals are profound, with one-third of patients requiring emergency medical care upon admission, and over half presenting with advanced ISS staging. In addition to the direct lethal impact on survival, an advanced disease stage also represents a financial burden to the health system due to prolonged hospitalization and expensive emergency medical interventions (24, 25).

In conclusion, our study presents the first-ever analysis of the diagnostic paths and delays experienced by Romanian MM patients. Our findings underscore the critical role of primary care physicians in reducing delays in diagnosis and highlight the need for targeted interventions to improve the diagnostic process.

Furthermore, educating family physicians about MM and offering support through the national health insurance platform could mitigate delays. Increasing awareness among the general population about MM symptoms and the importance of early detection is also crucial. Future research should focus on implementing interventions to streamline the diagnostic process and improve outcomes for MM patients in Romania.

This study has several limitations that should be considered when interpreting the findings. First, its single-center design may limit the generalizability of results Second, the relatively small sample size may impact the representativeness of the patient cohort analyzed. Furthermore, certain factors contributing to diagnostic delays, such as socioeconomic status and patient perspective, were not included in the data analysis. The study did not explicitly address possible recall bias, and the study period may not fully capture temporal trends.

## Data availability statement

The raw data supporting the conclusions of this article will be made available by the authors, without undue reservation.

## Ethics statement

The studies involving humans were approved by the Fundeni Clinical Institute's ethical committee board. The studies were conducted in accordance with the local legislation and institutional requirements. The participants provided their written informed consent to participate in this study.

## Author contributions

RI: Writing – review & editing, Writing – original draft, Project administration, Methodology, Investigation, Formal analysis, Data curation, Conceptualization. SoB: Writing – review & editing, Data curation, Supervision, SiB: Writing – review & editing, Data curation, IC: Writing – review & editing, Data curation, LZ: Writing – review & editing, Data curation. DC: Writing – review & editing, Supervision.
